# Vitamin D Status, *Cdx2* Genotype, and Colorectal Cancer Survival: Population-Based Patient Cohort

**DOI:** 10.3390/nu15122717

**Published:** 2023-06-12

**Authors:** Tafirenyika Gwenzi, Petra Schrotz-King, Ben Schöttker, Michael Hoffmeister, Hermann Brenner

**Affiliations:** 1 Division of Preventive Oncology, German Cancer Research Center (DKFZ), National Center for Tumor Diseases (NCT), Im Neuenheimer Feld 581, 69120 Heidelberg, Germany; 2 Medical Faculty Heidelberg, Heidelberg University, Im Neuenheimer Feld 672, 69120 Heidelberg, Germany; 3 Division of Clinical Epidemiology and Aging Research, German Cancer Research Center (DKFZ), Im Neuenheimer Feld 581, 69120 Heidelberg, Germany; 4 German Cancer Consortium (DKTK), German Cancer Research Center (DKFZ), Im Neuenheimer Feld 280, 69120 Heidelberg, Germany; 5 Network Aging Research, Heidelberg University, Bergheimer Straße 20, 69115 Heidelberg, Germany

**Keywords:** colorectal cancer, survival, vitamin D receptor, genotype

## Abstract

According to recent evidence, the prognostic value of Vitamin D (VitD) status for colorectal cancer (CRC) patients might be confined to patients with the GG genotype of *Cdx2*, a functional polymorphism of the VitD receptor gene. We aimed to validate these findings in a cohort of CRC patients. Post-operative serum 25-hydroxyvitamin D concentration was determined by mass spectrometry and *Cdx2* genotyping was performed from blood or buccal swabs using standard methods. Joint associations of VitD status and *Cdx2* with overall survival (OS), CRC-specific survival (CSS), recurrence-free survival (RFS), and disease-free survival (DFS) were assessed using Cox regression. For patients with GG genotype, adjusted hazard ratios (95% confidence interval) for the associations of sufficient compared with deficient VitD were 0.63 (0.50–0.78), 0.68 (0.50–0.90), 0.66 (0.51–0.86), and 0.62 (0.50–0.77) for OS, CSS, RFS, and DFS, respectively. These associations were weaker and not statistically significant for the AA/AG genotype. Interaction between VitD status and genotype did not reach statistical significance. VitD deficiency is an independent predictor of poorer survival, particularly for the GG *Cdx2* carriers, suggesting a potential role of VitD supplementation according to VitD status and genotype, which should be evaluated in randomised trials.

## 1. Introduction

The global disease burden of colorectal cancer (CRC) is among the highest of all cancers, accounting for more than 900,000 deaths each year [1]. Prognosis strongly depends on stage at diagnosis. However, a number of factors beyond stage may also be crucial for prognosis and, to the extent they are modifiable, may offer opportunities of targeted tertiary prevention. Vitamin D (VitD) deficiency and insufficiency among CRC patients has been associated with a significantly worse prognosis of these patients compared to those with adequate VitD status even after thorough adjustment for potential confounders [2,3,4,5]. This observation has raised the hypothesis that VitD supplementation may enhance prognosis of CRC patients.

A systematic review and meta-analysis of randomised VitD supplementation trials found a significant 30% reduction in adverse CRC outcomes with supplementation, which could have tremendous clinical value [6]. This finding is the more remarkable, as it was derived from studies in which VitD supplementation was provided regardless of initial VitD status, and other potential determinants of effectiveness of VitD supplementation. One particularly important factor in this context could be genetically determined vitamin D receptor (*VDR*) function.

In two large cohorts of CRC patients from the UK, a strong inverse association between 25-hydroxyvitamin D (25(OH)D), the best-established biomarker of VitD status, and CRC-specific survival was observed among patients with the GG genotype of rs11568820 (also known as *Cdx2*), a functional polymorphism located in the promotor region of the *VDR* gene, whereas this association was not seen among other patients [7]. Identifying patients most likely to benefit from VitD supplementation could pave the way for more effective, personalised VitD supplementation of CRC patients. The objective of this study was to thoroughly assess the joint association of serum 25(OH)D levels and the *Cdx2* polymorphisms with various survival outcomes in a cohort of CRC patients from Germany.

## 2. Materials and Methods

### 2.1. Study Details

We used data and serum samples of the DACHS-study, a population-based case-control study with long-term follow-up of patients with a first diagnosis of CRC recruited in south-west Germany between 2003 and 2021. The DACHS-study adheres to the guidelines of the Declaration of Helsinki. It was approved by the state medical boards of Baden-Württemberg and Rhineland-Palatinate, and the University of Heidelberg ethics committees (ethical code: 310/2001 approved on 6 December 2001). All participants provided written informed consent.

**Figure 1:**
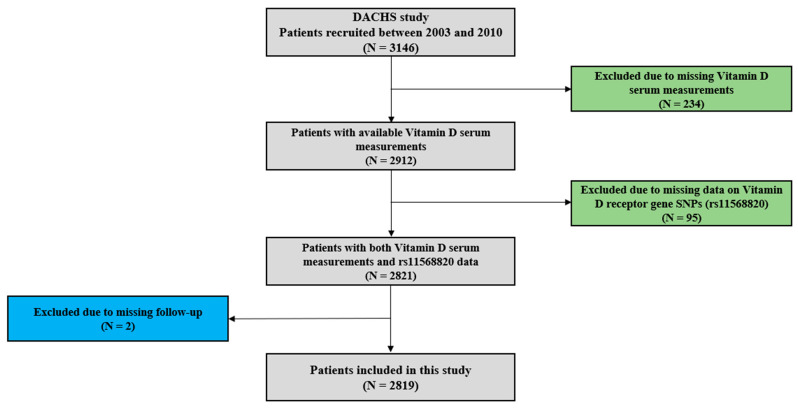
Patient selection flow chart.

Details of the DACHS-study have been previously reported [3,8,9,10,11]. In summary, patients who were eligible were identified from 22 participating clinics based on a first diagnosis of CRC (International Classification of Diseases, Tenth Revision [ICD-10] codes C18–C20). These patients were informed about the study shortly before or after surgery through clinicians or shortly after discharge by mail. Trained interviewers used standardised questionnaires to collect sociodemographic, lifestyle history, and medical information from study participants in personal interviews. Medical data on tumour stage, site, and therapy were obtained from hospital charts. Blood samples were collected after the personal interviews and serum aliquots were stored at −80 °C. Study participants were followed-up on therapy and health outcomes at 3-, 5-, and 10-year time points after diagnosis of CRC. Data on vital status were obtained from population registries and information on cause of death was obtained from health authorities, while details of recurrence and treatment were collected using standardised follow-up questionnaires. For the current study, 2819 patients with incident CRC and with both serum 25(OH)D measurements and *Cdx2* genetic polymorphism information available were included, who were recruited from 2003 to 2010 and followed-up for a median time of about 10 years (Figure 1).

### 2.2. Serum Vitamin D Measurements

Serum 25(OH)D measurements were conducted at the German Cancer Research Centre using High Performance Liquid Chromatography–Electro Spray Ionisation–Mass Spectrometry (HPLC–ESI–MS). The HPLC–ESI–MS method was standardised using the Standard Reference Material (SRM) 972a developed by the National Institute of Standards and Technology (NIST) [12]. VitD status was defined by the serum 25(OH)D cut-offs according to the United States-American Institute of Medicine as follows: Deficient (<30 nmol/L), insufficient (30 to <50 nmol/L), sufficient (≥50 nmol/L) [13].

### 2.3. Genotyping for Cdx2

Details for the determination of *VDR* gene single-nucleotide polymorphisms (SNPs) for this study have been reported elsewhere [14,15,16]. In summary, DNA was extracted from blood samples or, in exceptional cases in which blood samples were not available, from buccal swab samples of participants using standard methods [17]. Genotyping was conducted using Illumina array technologies (San Diego, CA, USA) and PLINK software (version 1.9) was used to extract information about *Cdx2* SNP genotypes AA, AG, and GG. For all analyses, *Cdx2* genotypes were categorised into a binary variable, with the rarer variants AA + AG as one category and GG as the other category.

### 2.4. Outcomes

Survival outcomes of overall survival (OS), CRC-specific survival (CSS), recurrence-free survival (RFS), and disease-free survival (DFS) were defined as death from any cause, death from CRC, recurrence of or death from CRC, and recurrence of CRC or death from any cause, respectively. Times of follow-up for survival outcome endpoints were counted in days from the date of CRC diagnosis to the date of experiencing the event. Patients were censored at a date when they were last known to have been alive or free of recurrence if they did not reach a specific endpoint.

### 2.5. Statistical Analyses

Descriptive statistics were used to analyse population characteristics. Survival analysis was performed using Cox proportional hazard (PH) models to calculate hazard ratios (HRs) for the individual and joint associations of predictors [serum 25(OH)D and *Cdx2* genetic variants] with survival outcomes (OS, CSS, RFS, and DFS). For joint associations, analyses were stratified by *Cdx2* as a binary variable. Two different adjustment models were used to evaluate the predictor–outcome associations. Model 1 analyses were adjusted for sex (male/female), age (30–59/60–69/70–79/>80 years), and season of blood collection (winter, spring, summer, autumn). Model 2 analyses were further adjusted for tumour detection mode (screening/other), cancer site (colon/rectum) and stage (I–IV) at diagnosis, chemotherapy use (yes/no), surgery (yes/no), history of cardiovascular disease (CVD) (yes/no), diabetes (yes/no), hypertension (yes/no), lifetime smoking exposure (never/<10/10–19/20–29/≥30 pack-years), body mass index (BMI) (normal/overweight/obese), physical activity (quartiles of average lifetime Metabolic Equivalent of Task hours per week), and time between diagnosis and blood collection (<1 month/≥1 month). Interactions between 25(OH)D as a continuous variable and *Cdx2* as a categorical variable with respect to survival were assessed by adding their product terms to model 2.

Cox PH model diagnostics were performed by evaluating interactions between time and covariates. Interactions between predictors and covariates were assessed by adding product terms to the regression models and evaluation of the corresponding Wald test statistics. Adjusted Kaplan–Meier (KM) survival curves were presented to assess survival outcomes according to serum 25(OH)D status and *Cdx2* genotype. All statistical tests were performed using R-statistical software (version 4.2) and two-sided test significance levels were set at *p*-values < 0.05 for all analyses.

## 3. Results

### 3.1. Description of Patient Characteristics

A total of 2819 patients were included in our analyses (Table 1). Nearly 60% of patients were male, and the median age at diagnosis for the cohort was 69 years (interquartile range: 62–76 years). More than 50% of the patients were diagnosed in stages I or II, and about 14% in stage IV of CRC. A majority of 59% of patients had a serum 25(OH)D in the deficient range. Close to 65% of patients had a GG genotype for *Cdx2*. In addition, serum levels of 25(OH)D did not differ according to *Cdx2* genotype. The proportion of patients with VitD deficiency was about 60% for all the three genotypes (GG, AG, and AA), while about 15% of patients had sufficient VitD status for all the genotypes (chi-square *p*-value = 0.64) (see Table 2). BMI interquartile range was 23.6–29.0 kg/m^2^ (median: 26.1 kg/m^2^) and about half of the patients were recruited within 30 days after primary diagnosis of CRC. After a median follow-up of 9.4 years, 1521 deaths were recorded and 798 of these were due to CRC.

### 3.2. Vitamin D Status and Survival

Associations between categories of serum 25(OH)D levels and survival outcomes are shown in Table 3. After controlling for sex, age, and season of blood draw, significantly better survival was seen for both patients with VitD insufficiency and those with sufficient VitD compared to VitD deficient patients. Although the associations between VitD status and survival outcomes were attenuated after adjustment for all covariates of interest, they remained statistically significant. Fully adjusted HRs (95% CI) for sufficient versus deficient VitD status were 0.71 (0.59–0.84), 0.76 (0.60–0.95), 0.79 (0.64–0.98), and 0.69 (0.58–0.82) for OS, CSS, RFS, and DFS, respectively. No significant interactions were observed between VitD status and categorical covariates, and thus subgroup analyses for these variables were not conducted.

### 3.3. VDR Cdx2 Locus Genotypes and Survival

Hazard ratios for the associations between *Cdx2* genotypes and survival outcomes are also presented in Table 3. After controlling for sex, age, and season of blood draw, no significant associations were observed between *VDR* genotypes and any of the survival outcomes. Similar results were also observed after adjustment for all covariates of interest. Fully adjusted HRs (95% CI) for AA/AG genotype versus GG genotype were 0.99 (0.88–1.11), 0.93 (0.80–1.09), 0.97 (0.84–1.11), and 0.98 (0.88–1.10) for OS, CSS, RFS, and DFS, respectively.

### 3.4. Joint Associations of Vitamin D Status and VDR Cdx2 Locus Genotypes with Survival

**Figure 2:**
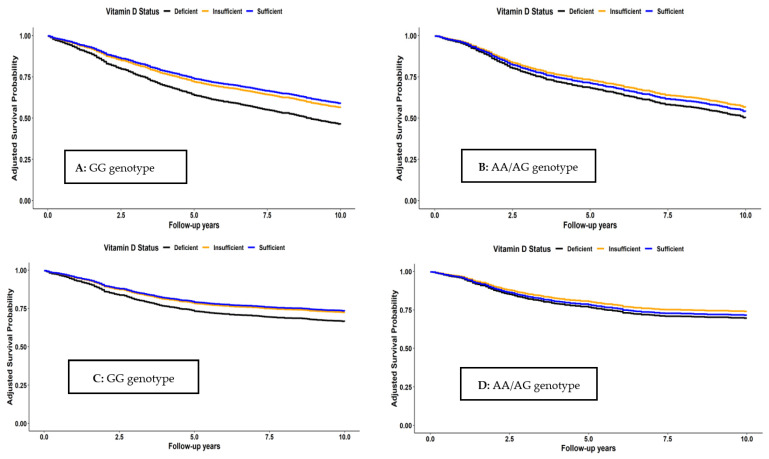

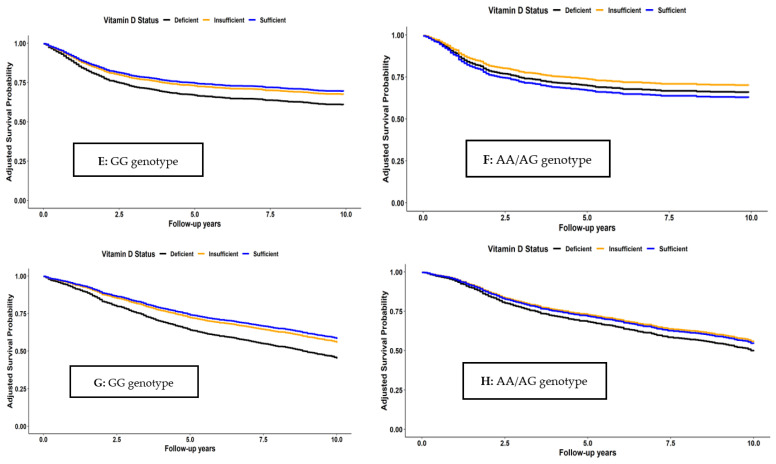
Overall (**A**,**B**), CRC-specific (**C**,**D**), recurrence-free (**E**,**F**), and disease-free (**G**,**H**) survival according to vitamin D status and *Cdx2* genotype.

Survival curves for the joint associations of VitD status and *Cdx2* genotypes are shown in Figure 2. Among those with GG genotype, survival was consistently higher for all survival outcomes in both patients with sufficient and insufficient VitD status than in those with VitD deficiency. In contrast, no clear associations were seen between VitD status and the survival outcomes among patients with AA or AG genotype. These patterns were also confirmed in the multivariable analyses that are shown in Table 4. For patients with GG genotype, adjusted hazard ratios (95% CI) for those with sufficient VitD (25(OH)D > 50 nmol/L) or insufficient VitD (25(OH)D between 30 and 50 nmol/L) compared to those with deficient VitD (25(OH)D < 30 nmol/L) were 0.63 (0.50–0.78) and 0.69 (0.56–0.84) for OS, 0.68 (0.50–0.90) and 0.71 (0.55–0.92) for CSS, 0.66 (0.51–0.86) and 0.73 (0.58–0.91) for RFS, and 0.62 (0.50–0.77) and 0.68 (0.56–0.83) for DFS, respectively. Trend analyses for VitD status were also significant for all outcomes among patients with GG genotype (*p*-trend < 0.01). In contrast, no consistent patterns and no significant trends were seen among those with AA/AG genotype, except for the outcome of DFS (*p*-trend = 0.04). However, tests for interaction between VitD and genotype with respect to the survival outcomes did not reach statistical significance.

## 4. Discussion

In this large cohort of CRC patients, patients with deficient VitD status had significantly worse survival than patients with VitD in the insufficiency range or with sufficient VitD. Although this association was clearly seen in the majority of patients who carried the GG genotype of rs11568820 (*Cdx2*), this clear pattern was not seen among those with AA/AG genotype. However, tests of interaction between VitD status and genotype did not reach statistical significance.

### 4.1. Vitamin D Status and Survival

Our results showed significant associations for VitD status and survival outcomes independent of other established prognostic factors, such as stage at diagnosis. Our findings of substantially worse survival among patients with serum levels of 25(OH)D in the VitD deficiency range (<30 nmol/L) compared to those with higher concentrations are in agreement with previous observational studies [4,7,18,19]. In our study, these associations were evaluated and consistently observed for all of the four assessed major survival outcomes. Although these associations from observational studies cannot be taken as evidence for causality, they are highly consistent with findings of a recent meta-analysis of RCTs, in which a 30% lower risk for CSS and progression-free survival (PFS) outcomes by VitD supplementation among CRC patients has been found [7]. Although the exact mechanism by which VitD improves survival in CRC patients is not clear, mechanistic studies have reported that calcitriol (the active form of VitD) acts in various ways via *VDRs* expressed on human cells to regulate transcription of genes involved in metastasis [20], cell proliferation [21], angiogenesis, cell differentiation, apoptosis, and DNA repair [22]. Calcitriol may also play a key role in suppressing cancer development and progression through immune-inflammatory modulation [23,24].

### 4.2. VDR Cdx2 Locus Genotypes and Survival

Our findings of null associations between *Cdx2* genotype and survival outcomes among CRC patients are consistent with previously reported null associations of VDR polymorphisms rs731236 (*Taq1*), rs2228570 (*Fok1*), *Cdx2*, and rs1989969 (*VDR-5132*) with OS and CSS in a significantly smaller (and partly overlapping) sample of CRC patients [14]. Whereas mixed results have been reported on the associations of *Cdx2* with CRC incidence, studies on prognostic outcomes are rather limited [25,26,27,28,29]. A meta-analysis published in 2016 found that the G-allele of the *Cdx2* gene was associated with a 12% higher risk for CRC [30]. This protective role of the *Cdx2* A-allele has previously been reported in a study that reported low risk of fractures among ethnic groups with higher A-allele frequencies. Higher frequency of the *Cdx2* A-allele was observed among study subjects of African descent, followed by Asian and lastly Caucasian groups (74%, 43%, and 19%, respectively) [31]. These findings may also suggest a possible interference of *Cdx2* with VitD status in the development and progression of cancer [32]. In another study by Ochs-Balcom et al., a strong and significant association between *Cdx2* and risk of colon cancer was only observed for people with low BMI or waist circumference [26]. Therefore, the authors postulated a modifying effect of adiposity on this association. However, no effect on modification was observed in our study for the association between *Cdx2* and survival outcomes by BMI.

The effects of VitD are mediated by the *VDR*, a member of the superfamily of nuclear receptors involved in regulation of a number of transcription genes. Consequently, cell response to VitD depends on the expression levels of the *VDR* [33]. CRC patients with low serum expression levels of *VDR* in a recent study have been reported to have poor prognosis compared to those with higher expression levels [34]. In addition, serum expression levels of *VDR* have been observed to be significantly lower for CRC patients than the general [35]. Future prognostic studies may need to consider both genotypes and serum expression levels of *VDR.*

### 4.3. Joint Associations of Vitamin D Status and Cdx2 Genotypes with Survival

Although tests for interaction between VitD status and *Cdx2* genotype with respect to survival did not reach statistical significance in our cohort, the pattern of strong inverse associations between VitD and mortality among those with the GG genotype, and absence of these associations among those with the AA/AG genotype is highly consistent with observations from two somewhat smaller CRC patient cohorts from the UK (n = 1687 and n = 1848, respectively) [7]. The weaker association between VitD status and survival in the entire cohort and among those with the GG genotype in our study may be due to different categorisations of VitD status (commonly employed standard categories in our study, tertiles in the UK studies) and somewhat more comprehensive confounder adjustment in our study (adjustment for 10 covariates including chemotherapy use, smoking, and physical activity).

The *Cdx2* SNP is located on the *VDR* gene at the 5′ end promoter region, and the polymorphism at the *Cdx2* locus plays a key role in calcium regulation. In a previous study among 261 Japanese women, the G-allele has been reported to reduce *VDR* transcription through the elimination of the *Cdx2* transcription binding site, while the A-allele was thought to upregulate *VDR* transcription [36]. In agreement with the results from the UK cohorts, results from our study, which, to the best of our knowledge, are the largest to investigate joint associations of VitD status and *Cdx2* genotype with survival outcomes in CRC patients, do not seem to support advantages of those with the AA/AG genotype with respect to CRC survival in these Caucasian populations.

### 4.4. Strengths and Limitations

Strengths of our study include the large sample size of patients who were recruited from all (more than 20) clinics providing CRC surgery in a defined study region and comprehensively followed with respect to all of the four common survival outcomes, comprehensive ascertainment of clinical and lifestyle factors, and adjustment for potential confounding factors. Nevertheless, residual confounding by unmeasured or not perfectly measured covariates cannot be ruled out and causality cannot be established in this observational study. Finally, our study included almost exclusively patients of Caucasian origin, and results may not be generalised to populations with different ancestries.

## 5. Conclusions

Findings from this large cohort of CRC patients provide further evidence that post-surgery VitD status is a strong, independent, and potentially modifiable prognostic factor for CRC patients, the association being particularly strong among those with the GG *Cdx2* genotype. Preliminary evidence from randomised trials supports suggestions that VitD supplementation may enhance prognosis of CRC patients. Given the dose-response relationship between VitD status and mortality outcomes, with worse survival being essentially restricted to those with VitD deficiency and (to a lesser extent) insufficiency, potential interventions, to be evaluated in further randomised trials, should focus on these groups of patients [3,37,38]. If and to what extent VDR genotypes or other potential effect modifying factors deserve additional consideration for potential personalised tertiary prevention should be further explored in future research.

## Figures and Tables

**Table 1:** Main characteristics of study population among 2819 colorectal cancer (CRC) patients.

Characteristic		N	%
Sex	Female	1136	40.3
	Male	1683	59.7
Age at diagnosis (years)	Median (IQR)	69 (62–76)	
	30–59	553	19.6
	60–69	914	32.4
	70–79	923	32.7
	80+	429	15.2
UICC Cancer Stage (TNM)	I	650	23.1
	II	879	31.3
	III	889	31.7
	IV	391	13.9
Cancer Site	Colon	1687	59.8
	Rectum	1132	40.2
Serum 25(OH)D	<30 nmol/L	1675	59.4
	30–49 nmol/L	695	24.7
	≥50 nmol/L	449	15.9
*Cdx2* genotype	AA	122	4.3
	AG	872	30.9
	GG	1825	64.7
Body Mass Index [kg/m^2^]	Median (IQR)	26.1 (23.6–29.0)	
	<25	1077	38.2
	25- < 30	1208	42.9
	≥30	534	18.9
Tumour detected by screening	Yes	658	23.4
	No	2161	76.6
Surgical treatment for CRC	Yes	2744	97.3
	No	75	2.7
Received chemotherapy	Yes	1287	45.9
	No	1532	54.1
History of cardiovascular disease	Yes	709	25.8
	No	2110	74.2
History of diabetes	Yes	521	18.5
	No	2298	81.5
History of hypertension	Yes	1445	51.3
	No	1374	48.7
Smoking exposure (lifetime pack-years)	Never	1280	45.7
	<10	501	17.9
	10–19	365	13.0
	20–29	278	9.9
	≥30	379	13.5
Alcohol consumption ^1^	None	818	29.4
	Low	1253	45.1
	High	710	25.5
Physical activity ^2^	Low	923	33.4
	Moderate	920	33.3
	High	917	33.2
School education [yrs.]	<9	1914	68.1
	9–10	470	16.7
	≥10	428	15.2
Late entry ^3^	≤1 month	1400	51.9
	>1 month	1300	48.1
Season of blood draw	Spring	780	27.7
	Summer	756	26.8
	Autumn	671	23.8
	Winter	612	21.7

Abbreviations: 25(OH)D: 25-Hydroxyvitamin D; IQR: Interquartile range; UICC: Union for International Cancer Control; MET-h: Metabolic equivalent task hours. ^1^ Sex-specific definitions (female: cut-off = 16 g ethanol/day; male: cut-off = 24 g ethanol/day); ^2^ According to MET-hours/week in the past 1-year as tertiles (low < 80; moderate 81- < 146.5; high >146.5); ^3^ Time between CRC diagnosis and blood collection. Season of blood draw (spring: ‘‘March to May’’, summer: ‘‘June to August’’, autumn: ‘‘September to November’’, winter: ‘‘December to February’’).

**Table 2:** Distribution of serum 25(OH)D level by *Cdx2* genotype.

Vitamin D Status	*Cdx2* Genotype		
	GG	AG	AA
Deficient	1100 (60)	502 (58)	73 (60)
Insufficient	443 (24)	220 (25)	32 (26)
Sufficient	282 (15)	150 (17)	17 (14)
Total	1825 (100)	872 (100)	122 (100)

Chi-square = 2.53; degrees of freedom = 4; *p*-value = 0.64; frequencies are presented as n (%).

**Table 3:** Individual associations of serum 25(OH)D concentration and *Cdx2* genotype with the different survival outcomes.

Survival Endpoint	Predictor		N/Events	Hazard Ratio (95% CI)	
				Model 1 *	Model 2 **
Overall	Serum 25(OH)D	<30 nmol/L	1673/1012	1.00 (ref)	1.00 (ref)
		30–49 nmol/L	695/317	0.67 (0.59–0.75)	0.73 (0.63–0.85)
		≥50 nmol/L	449/190	0.62 (0.53–0.72)	0.71 (0.59–0.84)
	*Cdx2* genotype	GG	1824/999	1.00 (ref)	1.00 (ref)
		AA or AG	993/520	0.95 (0.86–1.06)	0.99 (0.88–1.11)
CRC-specific	Serum 25(OH)D	<30 nmol/L	1657/542	1.00 (ref)	1.00 (ref)
		30–49 nmol/L	686/154	0.61 (0.51–0.73)	0.72 (0.59–0.89)
		≥50 nmol/L	442/102	0.61 (0.49–0.75)	0.76 (0.60–0.95)
	*Cdx2* genotype	GG	1806/534	1.00 (ref)	1.00 (ref)
		AA or AG	979/264	0.90 (0.78–1.05)	0.93 (0.80–1.09)
Recurrence-free	Serum 25(OH)D	<30 nmol/L	1662/612	1.00 (ref)	1.00 (ref)
		30–49 nmol/L	690/187	0.65 (0.55–0.76)	0.76 (0.64–0.90)
		≥50 nmol/L	444/119	0.62 (0.51–0.76)	0.79 (0.64–0.98)
	*Cdx2* genotype	GG	1811/603	1.00 (ref)	1.00 (ref)
		AA or AG	985/314	0.95 (0.83–1.09)	0.97 (0.84–1.11)
Disease-free	Serum 25(OH)D	<30 nmol/L	1661/1034	1.00 (ref)	1.00 (ref)
		30–49 nmol/L	690/331	0.67 (0.59–0.76)	0.73 (0.62–0.85)
		≥50 nmol/L	444/194	0.60 (0.51–0.70)	0.69 (0.58–0.82)
	*Cdx2* genotype	GG	1810/1026	1.00 (ref)	1.00 (ref)
		AA or AG	985/533	0.95 (0.86–1.06)	0.98 (0.88–1.10)

* Controlled for sex, age, and season. ** Additional adjustment for cancer stage, tumour site, tumour detection mode, chemotherapy, cardiovascular disease, diabetes, hypertension, smoking, body mass index, physical activity, and late entry.

**Table 4:** Joint associations of serum 25(OH)D concentration and *Cdx2* genotype with the different survival outcomes.

Survival Endpoint	*Cdx2* Genotype	25(OH)D	N/Events	Adjusted Hazard Ratio (95% CI) *	P_trend_
Overall	GG	<30 nmol/L	1099/683	1.00 (ref)	<0.001
		30–49 nmol/L	443/200	0.69 (0.56–0.84)	
		≥50 nmol/L	282/116	0.63 (0.50–0.78)	
	AA or AG	<30 nmol/L	574/329	1.00 (ref)	0.08
		30–49 nmol/L	252/117	0.77 (0.61–0.98)	
		≥50 nmol/L	167/74	0.85 (0.64–1.13)	
	P_interaction_	0.33			
CRC-specific	GG	<30 nmol/L	1091/370	1.00 (ref)	0.002
		30–49 nmol/L	439/102	0.71 (0.55–0.92)	
		≥50 nmol/L	276/62	0.68 (0.50–0.90)	
	AA or AG	<30 nmol/L	566/172	1.00 (ref)	0.24
		30–49 nmol/L	247/53	0.74 (0.52–1.05)	
		≥50 nmol/L	166/40	0.87 (0.59–1.29)	
	P_interaction_	0.88			
Recurrence-free	GG	<30 nmol/L	1090/411	1.00 (ref)	<0.001
		30–49 nmol/L	440/120	0.73 (0.58–0.91)	
		≥50 nmol/L	281/71	0.66 (0.51–0.86)	
	AA or AG	<30 nmol/L	572/200	1.00 (ref)	0.99
		30–49 nmol/L	250/67	0.79 (0.58–1.07)	
		≥50 nmol/L	163/47	1.13 (0.79–1.61)	
	P_interaction_	0.50			
Disease-free	GG	<30 nmol/L	1089/696	1.00 (ref)	<0.001
		30–49 nmol/L	440/209	0.68 (0.56–0.83)	
		≥50 nmol/L	281/121	0.62 (0.50–0.77)	
	AA or AG	<30 nmol/L	572/338	1.00 (ref)	0.04
		30–49 nmol/L	250/122	0.78 (0.62–0.99)	
		≥50 nmol/L	163/73	0.81 (0.61–1.08)	
	P_interaction_	0.40			

* Further adjustment for cancer stage, tumour site, tumour detection mode, chemotherapy, cardiovascular disease, diabetes, hypertension, smoking, body mass index, physical activity, and late entry. P_interaction_ was obtained by fitting a non-stratified full model with the interaction term for vitamin D serum level as a continuous variable and *Cdx2* as a binary variable.

## Data Availability

For ethical reasons, the data are not publicly available but may be availed upon request. For more details about the DACHS-study, please refer to the official website: dachs.dkfz.org/dachs/, accessed on 1 July 2021.
